# A large right side cardiac benign metastasizing leiomyoma of uterine origin as a rare cause of acute breathlessness

**DOI:** 10.1093/ehjcr/ytaf102

**Published:** 2025-02-25

**Authors:** Mahmoud Morsy, Mohammed Ellisy

**Affiliations:** St George’s University Hospital, NHS Foundation Trust, London SW17 0QT, UK; St George’s University Hospital, NHS Foundation Trust, London SW17 0QT, UK

A 34-year-old female with a history of hysterectomy for uterine fibroid with intravascular leiomyomatosis in 2019 presented with acute breathlessness (NYHA class IV) and ankle swelling. Clinical examination revealed raised jugular venous pressure and oedema of both lower limbs. Laboratory tests showed a negative D-dimer with elevated BNP of 1416. Chest radiography did not demonstrate any signs of lung congestion. Transthoracic echocardiogram revealed a large right-sided cardiac mass extending from the right atrium to the right ventricle across the tricuspid valve and circumferential pericardial effusion (*Figure 1A* and *B*). Due to the acute presentation, computed tomography of thorax, abdomen, and pelvis (CT-TAP) was performed, which revealed a 77 mm × 37 mm × 26 mm mass (*Figure 1C* and *D*), with no evidence of other organ involvement, residual or recurrent pelvic tumour. Following surgical review, the decision was made to operate, given the obstructive nature of the mass and the rapid progression of symptoms. The patient underwent excision of the mass with replacement of tricuspid valve with a bioprosthetic valve, due to severe tricuspid regurgitation after the mass excision (*Figure 1F*). The patient had an uneventful postoperative course and was discharged home after one week. During three- and six-month follow-up, the patient was asymptomatic. There was no evidence of residual mass in a two-month follow-up cardiac magnetic resonance imaging.

**Figure 1:**
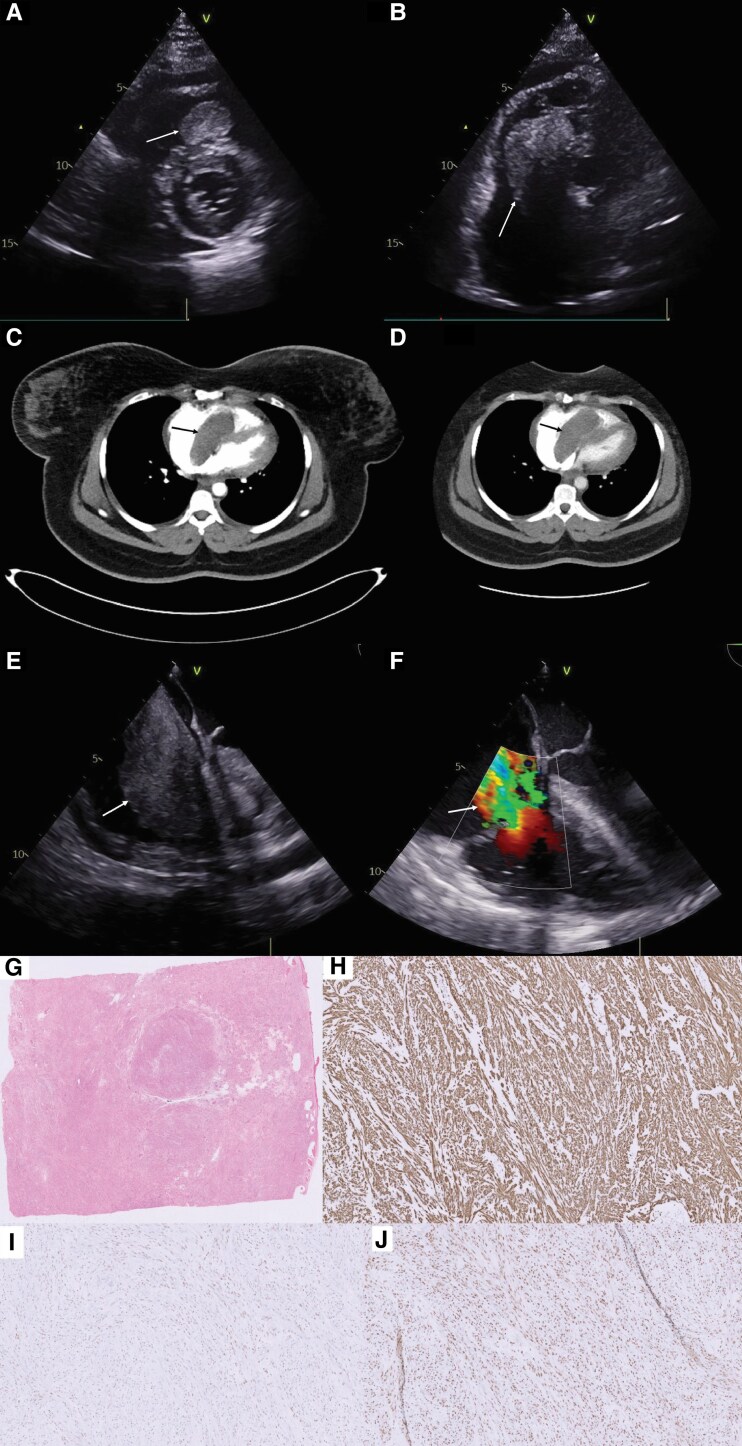
Bedside transthoracic echocardiogram (*A* and *B*), revealing the right-sided heart mass as the primary cause of patient’s presentation (arrows). CT with contrast (*C* and *D*), confirming the presence and site of the mass (arrows). Intraoperative transoesopageal echocardiogram showing the extension of mass from right atrium to right ventricle through the tricuspid valve (*E*), and severe tricuspid regurgitation after excision of the mass (*F*). Histopathology of the mass low power H&E (*G*), positive desmin (*H*), positive oestrogen receptors (*I*), and positive progesterone receptors (*J*), confirming that the mass is a leiomyoma.

Histopathological examination confirmed the diagnosis of leiomyoma, with the presence of oestrogen and progesterone receptors (*Figure 1G–J*). Multidisciplinary team discussion advised that the most probable diagnosis is a benign metastasizing leiomyoma (BML), given the rare incidence of primary cardiac leiomyomas and the history of intravascular leiomyomatosis. They advised to start hormonal therapy, to reduce the future recurrence of leiomyoma.

Resection of secondary cardiac tumours has been reported in the literature in situations where it poses haemodynamic compromise.^1^ Hormonal therapy is effective in reducing the recurrence of hormone-receptor positive BML but requires long-term adherence.^2^ In a published meta-analysis looking into 2277 patients who underwent tricuspid valve replacement with mechanical and bioprosthetic valves, there was no significant difference between the two groups in mortality, need for re-operation, and 5-year valve failure (see Supplementary material online, *Videos S1–S3*).^3^

## Supplementary Material

ytaf102_Supplementary_Data

## Data Availability

Data is available upon request from the corresponding author.

## Notes

**Consent:** The authors confirm that written consent for submission and publication of this manuscript including the images and associated text has been obtained from the patient in line with the COPE guidelines.

**Funding:** No funding was received.
